# Moral Disengagement and Generalized Social Trust as Mediators and Moderators of Rule-Respecting Behaviors During the COVID-19 Outbreak

**DOI:** 10.3389/fpsyg.2020.02102

**Published:** 2020-08-27

**Authors:** Guido Alessandri, Lorenzo Filosa, Marie S. Tisak, Elisabetta Crocetti, Giuseppe Crea, Lorenzo Avanzi

**Affiliations:** ^1^Department of Psychology, Sapienza University of Rome, Rome, Italy; ^2^Department of Psychology, Bowling Green State University, Bowling, OH, United States; ^3^Department of Psychology, Alma Mater Studiorum University of Bologna, Bologna, Italy; ^4^Department of Psychology, Salesian Pontifical University, Rome, Italy; ^5^Department of Psychology and Cognitive Science, University of Trento, Trento, Italy

**Keywords:** moral disengagement, big five, dark triad, COVID-19 outbreak, rule-respecting behaviors, social distance, moderation, mediation

## Abstract

In this study, we tested a theoretical model with moral disengagement, a mediator, and generalized social trust (GST), a mediator and a moderator of the relationship between personality traits and rule-respecting behaviors (i.e., social distancing and stay-at-home), during the coronavirus disease 2019 (COVID-19) outbreak in Italy. The data were collected on 1520 participants (61% males). General results are threefold: (1) moral disengagement mediated the relationship between emotional stability, narcissism, psychopathy, and social distancing; (2) among components of GST, trust in Government mediated the relationship between psychopathy and social distancing; trust in known others mediated the relationship between emotional stability, agreeableness, and Machiavellianism with total number of exits; trust in unknown others mediated the relationship of emotional stability, agreeableness, conscientiousness, and psychopathy with average daily number of exits; (3) GST moderated the indirect effect of personality traits on rule-respecting behaviors through moral disengagement. The theoretical and practical importance of these results is discussed.

## Introduction

Individuals living in Italy during the early months of 2020 experienced a sudden disruption and drastic change in their everyday life habits. Driven by the pandemic of the coronavirus disease 2019 (COVID-19) throughout the country, the Italian Government pursued and enforced the progressive restriction of individual freedom. First, Italians were required to practice social distancing, which is defined by the World Health Organization as avoiding close interpersonal contact by keeping a safe distance of 1 m (3 ft) from other people who are not from the same household in both indoor and outdoor spaces. Second, on February 23 of this year, the Italian Government announced other measures, including prohibiting individuals from both entering and exiting across the 14 most infectious municipalities, which were in the northern part of Italy (DPCM of February 23, 2020). These municipalities were declared “Protected Areas.” Then, on March 9, the Italian Government announced that the whole country was declared as “Protected Area” because the virus was spreading across Italy and was unrestrainable (Dpcm of March 9, 2020). Lastly, on March 22, the Government prohibited moving across municipalities by public or private transport, except for non-deferrable and proven work or health reasons (DPCM of March 22, 2020). These measures resulted in fundamental limitations on the movement of individuals, such as the Cancellation of all sports and cultural events, the closing of stores and restaurants, as well as the shutdown of schools and universities.

Both social distancing and the restrictions on personal freedoms have been strongly urged by medical experts and then imposed by the Government as an effective strategy to save the greatest number of lives possible. Thus, in name of a greater common good, people were asked to sacrifice one’s own personal rights in order to contribute to collective safety. As important as the goal may be, people often have difficulties in following rules, especially when they are imposed from the outside and are based on references to moral principles that are not always easy for everyone to understand (see Batson and Thompson, 2001).

In the early days of COVID-19 spreading, the percentage of active cases was scattered all around the country, being concentrated in the northern part of Italy (Task force Covid-19, 2020). Therefore, given the existence of relatively uninvolved areas, and despite the daily bleak news broadcasted by social media, the implications with respect to the rapid and worrisome development of the epidemic might have been underestimated by many individuals (see data by “Ministero dell’Interno” available at https://www.interno.gov.it/it/coronavirus-i-dati-dei-servizi-controllo). We also speculated that people who have initially strictly embraced the new rules may then have felt them unbearable to the point to (voluntarily or not) circumvent them. However disconcerting this may seem, our hypothesis is consistent with research revealing that people often violate the principles of civic behavior. This behavior occurs despite individuals being ethically committed and while continuing to profess the same principles, without incurring into any blame or feeling compelled to any kind of reparation (Bandura, 2016).

Studies on moral disengagement have indeed demonstrated that being able to acknowledge one’s moral obligations and to distinguish what is right from what is wrong does not always carry the will and capacity to behave accordingly (Caprara et al., 2014). By selectively disengaging one’s own sense of moral accountability, people may avoid taking full responsibility for the consequences of their actions that are in contrast with one’s own standards and values, and whose acknowledgment would imply an injury to one’s self-esteem. Yet, circumventing restrictions aimed to preserve public health, during the outbreak of a pandemic, may have high personal and societal costs, given the high level of infectivity of the COVID-19 (Task force Covid-19, 2020).

Moral disengagement is a malleable social cognitive orientation that depends both on individual dispositions, and on individual perceptions of the social context, such as generalized social trust (henceforth GST), which in turn can be defined as “the belief that most people can be trusted” (Uslaner, 2012, p. 7). Empirical studies have indeed reported strong associations among both individual normal personality traits (such as, agreeableness, conscientiousness, and emotional stability; Caprara et al., 2013, 2014) and the so-called dark personality traits (such as narcissism, Machiavellianism, and psychopathy; DeLisi et al., 2014; Fossati et al., 2014) and moral disengagement. Likewise, other studies have suggested that moral disengagement can be promoted or inhibited by perception of the social environment, such as GST (Hystad et al., 2014).

**FIGURE 1:**
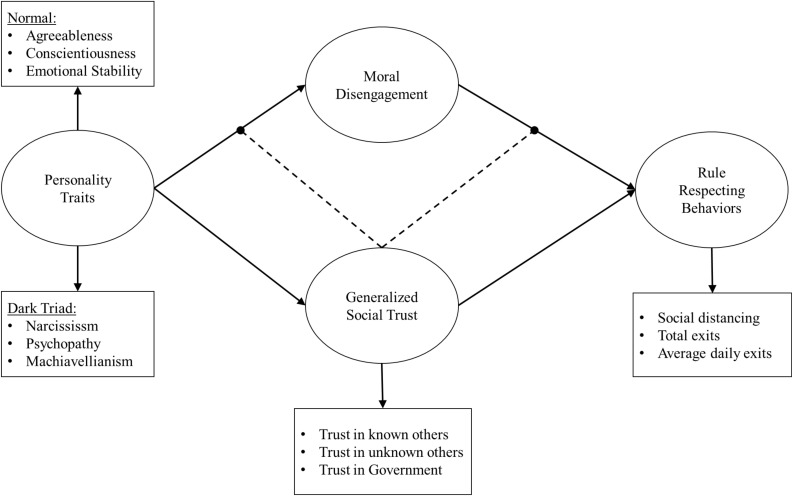
The hypothesized theoretical model.

Previous theoretical models have proposed that basic traits, proneness to moral disengagement, and perception of characteristics of the individuals’ social environment belong to different layers of the architecture of personality (Caprara et al., 2013, 2014). From this perspective, personality is conceived as a complex system including intrapersonal structures (such as basic traits) and characteristic adaptations (such as moral disengagement or GST). These structures and adaptations operate in concert but predict individuals’ behavior at different levels, distally and proximally, and thus to a different degree (see McCrae and Costa, 2008; but also, McAdams, 1995, for the notion of “level of analysis”). In this study, we proposed a theoretical model assigning to moral disengagement and GST the role of proximal predictors of rule-respecting behaviors (see Figure 1). These variables are conceived as rooted in personality traits that represent their dispositional basis and, thus, could mediate the effects of personality traits on rule-respecting behaviors. GST is further conceived as a moderator of the relationship between personality traits and moral disengagement and also of the association between moral disengagement and rule-respecting behaviors. Below, we present in detail the theoretical framework underlying our model and we explain in detail the role assigned to each variable.

### Moral Disengagement

When people engage in behaviors that contravene their personal standards, they usually experience negative affect produced by the state of cognitive dissonance engendered by the contrast between their actions and their principles (Bandura, 1990). To negate this unpleasant and often unbearable negative emotional state, people return to a series of cognitive strategies aimed to disengage from the moral sanctions of such behavior. Importantly, these maneuvers not necessarily happen after committing the transgression, but are often anticipatory and are aimed to reduce feelings of expected guilty or blame, and to make more likely and easier to legitimate committing the transgression in the pursuit of one’s self-interest (Bandura, 1990, 2016).

Self-sanctions can be decoupled from the enacting of detrimental conduct at four points (Bandura, 2016): (1) the behavior itself, (2) the locus of responsibility that is associated with the unethical behavior causing detrimental effects, (3) the harmful consequences, and (4) the recipient (or victim). At the behavior locus, mechanisms may act on the cognitive reconstruction of the behavior itself in order to transform harmful behavior in an acceptable behavior. The mechanism is aimed at social and moral purposes (moral justification), by labeling unethical actions with euphemistic language (euphemistic labeling), or by comparing individuals’ behavior with worse and more reprehensible deeds (advantageous comparison). Besides, people can disengage morally by covering or attenuating the agentic relation between their actions and the consequences (Bandura, 2016). People can also consider their behavior as dictated by social pressure or by a legitimate authority (displacement of responsibility) or by diffusing the responsibility for a joint action, making individual contribution undistinguishable (diffusion of responsibility). Turning to the outcome locus, individuals can resort to mechanisms that allow them to minimize or distort the consequences of their actions, or to ignore the blameful effects of their behavior. Finally, mechanisms at the recipient locus allow people to withdraw empathetic and sympathetic feelings for the victims by considering them responsible for their condition and deserving harm and punishment (attribution of blame) or by depersonalizing and dehumanizing them (dehumanization; Bandura, 2016).

A seminal work by Bandura and colleagues provides support for the disinhibitory effects of moral disengagement mechanisms on harmful and aggressive behavior (Bandura, 1990). Moreover, recent studies expanded this line of research including violation of social or organizational rules and norms (Detert et al., 2008). For instance, moral disengagement has been linked to organizational corruption and corporate transgressions (Bandura et al., 2000), support for war and military actions (McAlister et al., 2006), propensity for business choices that can harm the environment (Shepherd et al., 2013), and harmful civic behaviors and shirking civic duties (Caprara et al., 2009). With regard to the latter, this aspect of moral disengagement has been named civic moral disengagement, and it refers to the social cognitive mechanism that allows the individual to justify his or her actions that are reprehensible and damaging to social safety (Caprara et al., 2009). In the present study, we focalized on this type of civic moral disengagement and we expected it to play a key role in explaining low rule-respecting behaviors.

### Generalized Social Trust

Considering social trust can increase our understanding of moral disengagement as a social cognitive orientation that can be shaped by the nature of the external social contexts in which individuals live (Hystad et al., 2014). Accordingly, moral disengagement can be triggered in morally permissive environments, where the self-interest of single individuals is put before the obedience to societal values (Shu et al., 2011; Hystad et al., 2014). Theory of social norms suggests that the behavior of individuals largely depends on their perception of the quantity and frequency of that specific behavior conducted by others (Scholly et al., 2005). Such perceptions of the behaviors performed by others in a specific social context – perceived descriptive norms – play a significant role in the behavioral decisions of individuals (Rimal et al., 2005). That is, those who accept the regulations or social norms face the burden based on the social distancing rules or the stay-at-home order. This is because the perceived normativity of social distancing and stay-at-home orders would give to a group member the social proof that those around them will likely behave respecting social distancing, leading them to behave in the same way (Rimal et al., 2005). In contrast, others who violate these norms may promote an egoistic climate that may trigger individuals’ moral disengagement mechanisms (Moore and Gino, 2013).

For many instances, the construct of general social trust carves the notion of social reciprocity at its joints (see Rahn and Transue, 1998). GST can be conceptualized as a “standing decision” to give most people – even those whom one does not know from direct experience – the benefit of the doubt (Yamagishi and Yamagishi, 1994). Theoretical perspectives on GST often start from the premise that GST is “…the root cause of much of what is valued in today’s societies” (Oskarsson et al., 2012, p. 21). This principle has found support in empirical studies linking GST to several positive outcomes for the members of the society (Putnam, 1993; Sullivan and Transue, 1999; Nannestad, 2008; Uslaner, 2012; Dinesen and Bekkers, 2017), and a clear link has been established between GST and collective action (Putnam, 1993; Sønderskov, 2011). According to Sønderskov (2011), “generalized social trust enhances cooperation because most humans tend to cooperate when they expect others to do the same” (p. 66). Most importantly, from our perspective, GST is expected to put a constraint on the pursuing of unethical behaviors, while increasing group solidarity and cohesion (Devine, 1972). In fact, people who deem others as trustworthy are more likely to follow moral values and less likely to engage in antisocial behaviors (such as lying, cheating, or stealing; Rotter, 1980).

In this paper, following recommendations by the Organization for Economic Co-operation and Development (OECD; Gonzales and Smith, 2017; but see also Naef and Schupp, 2009; Uslaner, 2012), we explored the value of three important components of GST, namely, trust in people you know, trust in people you do not know, and trust in Government. Several authors advocated that distinguishing the first two components of GST is important for a meaningful understanding of the construct (e.g., Hardin, 2004; Delhey et al., 2011). Indeed, whereas people might highly trust their family members or close friends, they might have lower trust in someone they do not know personally. Trust in Government is another component of this conceptualization of GST (Gonzales and Smith, 2017; see also Hardin, 2004; Delhey et al., 2011) and a crucial ingredient of societal functioning (Marien and Hooghe, 2011; Jahromi et al., 2012).

Generally speaking, trust in Government can be seen as a form of diffuse support that a political system receives from its environment (Marien and Hooghe, 2011). Given the exceptional and unexpected nature of the rule enforced by the Italian Government in reaction to the COVID-19 outbreak, political trust can play an important role in fostering rule-respecting behaviors. Indeed, trusting citizens are more likely to perceive political decisions as being legitimate than distrusting citizens, even if these decisions are unfavorable to their own particular interests (Rudolph and Evans, 2005). Distrusting citizens, on the other hand, are more likely to calculate the costs and benefits of compliance, and this might lead to rule-breaking behaviors (Tyler, 1990). Within this framework, diffuse political trust can be considered an individual attribute essential resource to governing a society effectively.

The above reasoning led us to conceive two general hypotheses. The first is related to the role of components of GST as buffers (and thus as a “moderator”) of the expected negative relationship between moral disengagement and rule-respecting behaviors. The more individuals perceive social distancing and staying at home as common goals collectively pursued along with all other fellow citizens, the more they will try to respect them. The second points to a role of the components of GST as promotors of rule-respecting behaviors (i.e., “as a direct predictor”). Likewise, the more citizens trust the decisions enforced by their government, the more they may be expected to respect them, and the more they will consider it morally unacceptable to disrespect them (Marien and Hooghe, 2011). Therefore, engaging in rule-respecting behaviors will be perceived as normative, while deviance will be deemed as highly dysfunctional because of the expected high frequency of the first compared with the second.

### Personality Traits

Moral disengagement and social and political trusts are not fixed quantities possessed in the same way by all the individuals belonging to a certain social context. Previous studies have indeed shown that variation in individuals’ responses to moral disengagement can be ascribed to basic individual differences in ways of thinking, feeling, and behaving, namely, personality traits (Caprara et al., 2013, 2014). Likewise, according to the *dispositional perspective* (Dinesen and Bekkers, 2017), “trust is considered a downstream consequence of proximate dispositions such as personality traits” (Dinesen and Bekkers, 2017, p. 79). Several empirical studies have supported this notion, indicating an existing link between personality traits and GST (Hiraishi et al., 2008; Sturgis et al., 2010; Oskarsson et al., 2012; Merolla et al., 2013). Recently, Weinschenk and Dawes (2018) reported that genetic factors account for 64.40 and 59.73% of the observed (statistically significant) correlation between social trust and, respectively, (1) agreeableness (*r* = 0.25; 95% CI = 0.22, 0.28), a trait associated with cooperation and relating positively to others (Graziano and Eisenberg, 1997; DeYoung, 2015), and (2) neuroticism (or low emotional stability) (*r* = −14; 95% CI = −0.17, −0.11), a basic predisposition linked to the experience of negative emotions or mood, such as anxiety, sadness, discontent, and inadequate feelings (McCrae and Costa, 2008). Finally, there is evidence that the above personality traits are associated with trust in Government (Schoen and Schumann, 2007; Mondak and Halperin, 2008). Importantly, agreeableness, emotional stability, and conscientiousness are associated with moral disengaging tendencies (Caprara et al., 2013, 2014). Likewise, traits characterized by diligence, dutifulness, and hardworking, as well as the tendency to follow rules and resist immediate gratification to pursue longer-term goals (DeYoung, 2015) have been found to be related to moral disengaging tendencies (Caprara et al., 2013, 2014).

Based on prior studies, we conceptualized that individual differences in moral disengagement and GST are expressions of a tendency to self-indulgency fostered by low emotional stability and lack of agreeableness and conscientiousness (Caprara et al., 2013, 2014). In other words, we predicted that rule-respecting behaviors are only indirectly connected to basic traits, via the mediation of moral disengagement and of GST.

Other studies have pointed to the relationship of other dysfunctional personality traits, such as (a) narcissism, a trait capturing a lack of modesty, high interpersonal dominance, selfishness, and a need for attention (Campbell and Miller, 2011); (b) Machiavellianism, a trait characterized by a lack of empathy, by manipulation and the use of exploitative tactics, amorality, and a cynical worldview (Gunnthorsdottir et al., 2002); and (c) psychopathy, a trait distinguished by its callousness and un-sentimentality, apathy, impulsiveness and lack of self-control, irresponsibility, low affect, and the absence of remorse and guilt (Lynam and Derefinko, 2005). Paulhus and Williams (2002) introduced the term *dark triad* to refer to these socially aversive personality dimensions. These three traits share a common core of adversity toward others, amorality, and disregard for rules (Fossati et al., 2014). In fact, past research has shown that dark triad traits are related to a wide range of negative outcomes, such as interpersonal exploitation, deviant behaviors, aggression, and delinquency (see O’Boyle et al., 2012; Muris et al., 2017; for meta-analyses). Thus, based on the above findings, we hypothesized a negative association among dark traits and GST.

Previous studies have reported associations among the dark triad traits and moral disengagement (O’Kane et al., 1996; Shulman et al., 2011; DeLisi et al., 2014; Fossati et al., 2014; Sijtsema et al., 2019). Importantly, the study by Fossati et al. (2014) suggested that moral disengagement is one of the common features of pathological narcissism and psychopathy. These results are understandable by referring to the nature of the dark traits. Shulman et al. (2011), for example, maintained that psychopathic youth may be more prone to justifying antisocial conduct, given that they are less prone to experience moral emotions such as shame and guilt (see DeLisi et al., 2014). Narcissists are more likely to view others as either stupid or evil, or idolize them: Thus, they may perceive less morally reprehensible to exploit or abuse others. Finally, the psychological processes characterizing Machiavellianism are conceptually highly similar to the mechanisms of moral disengagement (see Fossati et al., 2014).

### The Present Study

With the aim to furthering our understanding of the mechanism fostering rule-respecting behaviors during the COVID-19 pandemic, we tested a theoretical model assigning to moral disengagement the role of the proximal predictor of two important classes of rule-respecting behaviors: namely, social distancing and stay-at-home. In this model, moral disengagement was further assigned the role of the mediator of the relationship between basic normal (i.e., agreeableness, emotional stability, and conscientiousness) and dark (i.e., narcissism, Machiavellianism, and psychopathy) personality traits and rule-respecting behaviors. Moreover, we assigned to GST the role of mediator of the relationship between personality traits and rule-respecting behaviors and the role of moderator of the relationships between (1) moral disengagement and rule-respecting behaviors and (2) personality traits and rule-respecting behaviors. Moreover, we considered a set of important covariates expected to correlate with rule-respecting behaviors during the COVID-19 pandemic. Below, we explain the theoretical arguments and reasoning underlying our hypotheses that were preregistered^1^.

Summarizing, in the present study, we tested the conceptual model represented in Figure 1. According to the model, moral disengagement and GST mediate the relationship between personality traits and rule-respecting behaviors. The above statement is in line with the different role assigned by our theoretical model to moral disengagement, which can hardly be viewed as a trait, as it does not concern pattern of thought, affect, and behavior. Whereas we did not dismiss that moral disengagement can be very stable across time, we therefore treat it as a contextual adaptation resulting from the expression and the interaction of individuals’ traits within their life environment (see Kish-Gephart et al., 2014). Positing basic traits and moral disengagement into different layers of our personality architecture assign them a different predictive power with respect to enacted behaviors, suggesting a predictive advantage for moral disengagement.

Finally, we expected that GST moderated the hypothesized relationship of traits with moral disengagement, so that the higher GST, the lower the impact of basic traits. Moreover, by increasing the moral value of rule-respecting behaviors, we also expect that GST will lessen the negative relationship between moral disengagement and rule-respecting behaviors. More formally, we stated that, at high levels of GST: (1) the expected negative association between agreeableness, emotional stability, conscientiousness, and moral disengagement will be stronger; (2) the expected positive relationship between narcissism, Machiavellianism, psychopathy, and moral disengagement will be stronger; and (3) the hypothesized negative relationship between moral disengagement and rule-respecting behaviors would be weaker.

We included and adjusted for several covariates potentially linked to the outcomes and to the mediating variables. Gender, age, and marital status were included because they were associated with moral disengagement and GST in previous studies (Jonason et al., 2012; Riedl and Javor, 2012; Gini et al., 2014). We also included covariates more directly linked to pandemic distress, such as the geographic area of residence (the northern parts of the country were more plagued than the center, the south and the islands), the number of infected people in the town, and the perceived contagion risk. Home size and number of roommates were included as a measure of economic distress.

## Materials and Methods

### Power Analysis

Participants for this investigation were drawn from the “Orientation toward Common Good” study (OCG-COVID-19). This study was, to the best of our knowledge, the first one examining the link between personality traits, moral disengagement, GST, and rule-respecting behaviors directly related to management of the COVID-19 pandemic. Therefore, the minimum effect size on which to base a power analysis was not clear when we were planning the research. Thus, we settled to achieve a sample size useful to attain an 80% power to detect effects equivalent to one fifth (i.e., *r* = |0.05|) the average effect of 0.20 usually found in psychological research (see Paterson et al., 2016). Accordingly, we planned to collect at least 1000 participants, which granted us an approximately 80% power, and was about two and a half higher than the size of 250 estimated to be the point where effects “stabilize” (Schönbrodt and Perugini, 2013). All data, script, and a detailed online appendix are available from https://osf.io/dkbpj/?view_only=196be18f7b454e0a84799ebdb91129f3.

### Sample

A total of 2377 individuals participated in the study. Of them, 1520 (64%) provided useful data on the measures considered in the present paper (subjects were excluded if they did not finish the entire questionnaire, or if they failed to fill out two check attention questions). No differences were found between included and excluded people on basic demographic characteristics. Participants (61% males) had an average age of 34.62 (*SD* = 16.15). About 75% of the sample were single, about 24% were married, about 5% were divorced, and the remaining 1% were widowed. The geographic distributions were north 10%, center 79%, south 8%, and islands 4%.

### Procedure

The OCG-COVID-19 is a collaborative national study promoted by researchers rooted in four Italian Universities. It was designed in order to understand the psychological determinants of individuals’ civic behaviors and adjustment at the social changes determined by the Government response to the COVID-19 outbreak. The study has been approved by a Sapienza Internal Review Board (“p.n. 0000576”) and was conducted from March 22 to April 6, after the issuing of the decree “Dm 25/3/2020” that declared all Italy as a “protected zone.” Participants were recruited using multiple methods such as participants’ list, advertising on national press, posts on social network, and snowball technique. Individuals were first contacted, invited to take part in the study, and briefly acquainted with its general aims. Individuals who accepted to participate received a link to fill out the questionnaire online. When participants filled out the questionnaire, they provided information about their geographic location. This information was then used for assigning them to 1 of the 85 cities involved in the study and for linking them with the total number of contagions observed for that day in their city, by using data provided by the state agency in charge of the emergency^2^. These data were then included in the analyses.

### Measures

In order to reduce respondents’ burden, we used short versions of the study measures. The validity and reliability of these scales have been extensively shown in previous publications. In this vein, agreeableness (ω = 0.25), emotional stability (ω = 0.45), and conscientiousness (ω = 0.57) were assessed with the Ten-Item Personality Inventory (TIPI; Gosling et al., 2003). The dark triad, namely, Machiavellianism (ω = 0.87), psychopathy (ω = 0.70), and narcissism (ω = 0.82), were assessed with the 12-item Dark Triad Dirty Dozen (DTDD; Jonason and Webster, 2010). Moral disengagement (ω = 0.74) was assessed using a reduced eight-item version of the Civic Moral Disengagement scale (CMD; Caprara et al., 2009 but see the Online Supplementary Material for detail about is development). The three components of GST, namely, “trust in known others” (ω = 0.71), “trust in unknown others” (ω = 0.91), and “trust in Government” (ω = 0.83), were assessed in agreement with OECD standards (Gonzales and Smith, 2017), and full details on how these constructs are measured is offered on the Online Supplementary Material. Rule-respecting behaviors were assessed with three items devised to assess compliance with the “social distancing rule” (ω = 0.58), one item asked about the total number of exits from home since the issuing of the stay-at home order, and another asked about the average daily number of exits from home. The Online Supplementary Material offers full details about the psychometric properties of these measures (see Supplementary Tables S1–S5). Gender (0 = female, 1 = males), age (in years), marital status (contrast coded: reference category = “single”), number of roommates, home size (in squared meters), geographic area (contrast coded: reference category “north”), number of infected people (obtained as explained above), perceived risk of infection (from 0 = no risk, to 100 = certainty), social activity (computed by averaging the frequency with which participants engaged in social activities before the COVID-19 outbreak, ω = 0.70; see Supplementary Table S6), and the day in which the questionnaire was filled out were included in the model as covariates.

### Strategy of Analysis

We tested our hypotheses following two successive but linked steps. In step 1, we examined the mediating role of moral disengagement and GST components, on the relationship between personality dispositions and the three rule-respecting behaviors (i.e., “social distancing,” “total number of exits from home since the beginning of quarantine,” and “daily exits from home”). We present results as obtained by the stepwise procedure introduced by Baron and Kenny (1986). However, in testing mediation, we focused on the significance of the indirect effect of traits on rule-respecting behaviors through moral disengagement and GST, as evaluated by procedures outlined by MacKinnon et al. (2002). The values for the upper and lower confidence intervals (CIs) for indirect effects were tested using the Monte Carlo method for assessing the mediation CI method (Hayes and Scharkow, 2013) with 20,000 replications.

As a second step, we tested if components of GST moderated the relation between personality traits and (1) moral disengagement or (2) the outcomes. Mediation and moderation hypotheses were integrated by using procedures devised by Preacher et al. (2007), which require the empirical test of two models. The first model investigates whether there is evidence of a significant moderation of the relation between personality traits (i.e., the independent variable) and moral disengagement (i.e., the mediating variables), by the different components of GST. The second model tested the statistical significance of the moderation of the mediational relationship (i.e., the indirect effect of personality traits on rule-respecting behaviors operated through moral disengagement) operated by GST.

All analyses were conducted in the *R* 3.6.3 statistical program (R Development Core Team, 2016). Multiple linear regression was used for estimating models predicting all mediators and the outcome “social distancing.” “Total number of exits from home” and “average daily number of exits from home” were count variables and showed overdispersion, as attested by high level of skewness (5.79 and 9.47, respectively). Thus, to appropriately model the relationship between the predictors and the frequency of exits, we used a Quasi-Poisson regression. The basic Poisson regression model assumes that the conditional variance is equal to the conditional mean, a condition that is seldom met in empirical research. In our data, the observed variances (i.e., 82.83 and 1.17) were far higher than their respective means (i.e., 5.07 and 0.39). Thus, we used a Quasi-Poisson model, which assumes that the variance is a linear function of the mean and thus resulted in being more adequate (Cameron and Trivedi, 1998; Osgood, 2000). Model fit was assessed by using *R*^2^ with reference to Cohen’s (1992) criteria of *R*^2^ equal to 0.26 as substantial, 0.13 as moderate, and 0.02 as weak (see also Ellis, 2010).

Before being entered in the models, all first-order terms were centered around the sample’s grand mean: this helps to eliminate non-essential multicollinearity and improve the interpretation of coefficients in models including multiplicative (interaction) terms (see Aiken and West, 1991). Their values can be interpreted as the observed change in the outcome variable when the independent variable moves one unit above or below the mean. To simplify the interpretation of terms in the Quasi-Poisson regression model, we exponentiated all coefficients. The resulting values represent the change in number of exits for each unit increase in the predictor.

## Results

### Zero-Order Correlations

Table 1 presents zero-order correlations among all the study variables. The average correlation of moral disengagement with agreeableness, emotional stability, and conscientious was moderately low (*r*_*m*_ = −0.16), with the highest one observed with agreeableness (*r* = −0.20). The correlations between moral disengagement and the dark traits were instead moderately high (*r*_*m*_ = −0.30), especially that with psychopathy (*r* = −0.36). GST and moral disengagement were moderately low correlated (*r*_*m*_ = −0.16), with higher correlations observed for trust in Government (*r* = −0.22). The correlations between GST and normal (*r*_*m*_ = 0.10) and dark (*r*_*m*_ = 0.12) personality traits were moderately low with higher values for the relationship of “trust in known others” with conscientiousness and Machiavellianism, respectively.

**TABLE 1:** Zero-order correlations.

	(1)	(2)	(3)	(4)	(5)	(6)	(7)	(8)	(9)	(10)	(11)	(12)	(13)	(14)	(15)	(16)	(17)	(18)	(19)
1. Sex	1																		
2. Age	−0.09**	1																	
3. N-Cohabitants	−0.06*	–0.04	1																
4. Home size	–0.03	0.03	0.15**	1															
5. N-Infected	−0.05*	0.04	–0.00	0.01	1														
6. P. Infection risk	0.05*	0.09**	−0.07**	–0.02	0.02	1													
7. Social activity	0.00	−0.41**	−0.14**	–0.02	0.00	–0.02	1												
8. Agreeableness	–0.01	0.17**	0.02	–0.01	0.00	–0.01	−0.13**	1											
9. Conscientiousness	0.05*	0.15**	–0.01	0.03	0.01	–0.03	−0.10**	0.21**	1										
10. Emotional stability	−0.25**	0.24**	0.02	0.04	0.02	−0.05*	−0.09**	0.33**	0.26**	1									
11. Narcissism	−0.09**	−0.23**	–0.01	–0.02	0.00	–0.04	0.13**	−0.26**	−0.17**	−0.18**	1								
12. Psychopathy	−0.17**	−0.31**	–0.04	–0.02	–0.02	−0.08**	0.15**	−0.34**	−0.19**	−0.09**	0.35**	1							
13. Machiavellianism	−0.13**	−0.27**	–0.01	–0.02	–0.01	−0.05*	0.18**	−0.27**	−0.27**	−0.17**	0.49**	0.47**	1						
14. Moral disengagement	−0.10**	−0.15**	0.00	0.00	–0.04	–0.01	0.09**	−0.20**	−0.16**	−0.13**	0.25**	0.36**	0.32**						
15. Trust k.o.	0.02	0.15**	0.13**	0.04	0.05*	0.02	0.02	0.17**	0.10**	0.18**	−0.14**	−0.18**	−0.21**	−0.19**	1				
16. Trust u.o.	−0.06*	0.23**	0.03	0.03	0.01	0.06*	0.00	0.11**	0.00	0.13**	−0.06*	−0.14**	−0.08**	−0.08**	0.39**	1			
17. Trust go.	0.00	0.09**	0.08**	0.03	–0.01	0.01	–0.02	0.09**	0.06*	0.08**	−0.06*	−0.11**	−0.06*	−0.22**	0.31**	0.28**	1		
18. Social distancing	0.13**	0.00	–0.03	0.02	0.02	–0.03	–0.01	0.11**	0.12**	0.02	−0.05*	−0.12**	−0.10**	−0.15**	0.09**	0.00	0.1	1	
19. Total exits	−0.12**	0.16**	−0.10**	–0.02	0.05*	0.12**	−0.05*	0.02	0.03	0.11**	–0.02	0.00	–0.02	0.00	−0.05*	0.04	−0.06*	−0.08*	1
20. Daily exits	−0.10**	0.12**	−0.08**	–0.02	0.00	0.09**	0.00	0.00	0.00	0.04	0.00	0.01	0.01	0.05*	–0.04	0.01	−0.08*	−0.12**	0.39**
Means	–	34.60	2.94	162	1348	35.90	2.18	5.12	5.29	4.45	2.51	1.88	1.69	1.79	3.38	2.30	2.94	3.58	5.07
SD	–	16.10	1.93	1170	969	24.00	0.50	1.10	1.16	1.40	0.93	0.74	0.78	0.56	0.60	0.76	0.80	0.44	9.10

Trust k.o., trust in known others; Trust u.o., trust in unknown others; Trust go., trust in Government. The home size values refer to the entire living space in which people can move freely (e.g., garden, terrace, etc.). *p < 0.05, **p < 0.01.

Considering the outcomes of interest in this study, we found (1) significant and moderately low positive correlations of “social distancing” with agreeableness, conscientiousness, and “trust in known others,” and moderately low, but negative, correlations with moral disengagement, psychopathy, and narcissism; (2) the “total number of exits from home” were positively and moderately low related with emotional stability. This variable also showed low correlations with “trust in known others” and social activity. Number of “daily exits” showed low correlations with moral disengagement. Finally, social distancing showed moderately low correlations with both the total and average daily exits from home, and these two latter variables resulted in moderately high correlation.

### Step 1. Mediator Models

#### Moral Disengagement

##### Hypothesized results

**FIGURE 2:**
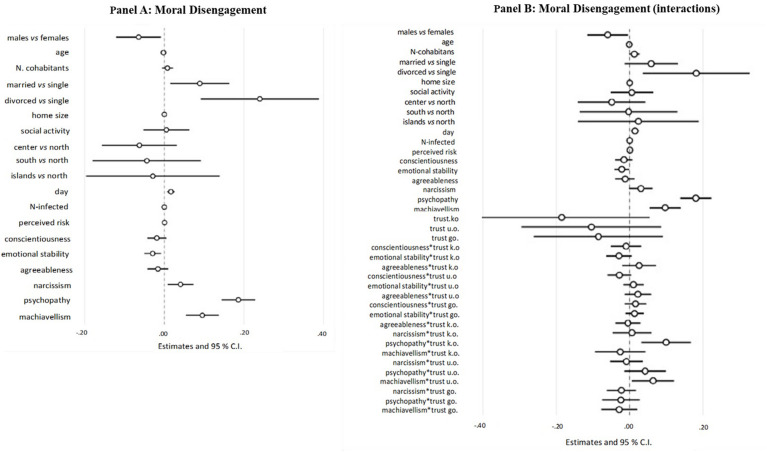
Results from the moral disengagement mediator model. The spheres represent regression coefficient estimates and the bars represent 95% confidence intervals (CIs); Trust k.o., trust in known others; Trust u.o., trust in unknown others; Trust go., trust in Government.

As shown in Figure 2A, moral disengagement was significantly and negatively predicted by emotional stability but positively and significantly predicted by narcissism, psychopathy, and Machiavellianism.

##### Not hypothesized results

We found significant higher levels of moral disengagement for males than for females and for single than for married or divorced respondents. Interestingly, we found that level of moral disengagement showed a significant tendency to increase with the passing of days and to be negatively associated with the number of infected people.

##### Model fit

The model explained a significant [*F*(19, 1500) = 18.87, *p* < 0.001] and moderately high proportion of variance (*R*^2^ = 0.19).

##### Hypothesized moderations

**FIGURE 3:**
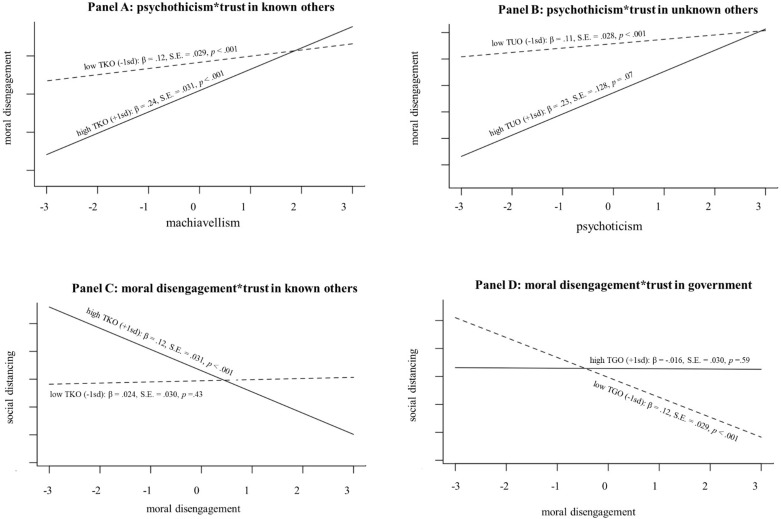
Graphical representation of significant interactions. **(A)** Psychothicism*trust in known others TKO. **(B)** Machiavellianism*trust in unknown others. **(C)** Moral disengagement*trust in known others. **(D)** Moral disengagement*trust in government. TKO, trust in known others; TUO, trust in unknown others; TGO, trust in Government.

Figure 2B presents results for the model testing the moderation of GST on personality traits in the prediction of moral disengagement. This model was significantly better than the former [*F*(21, 1479) = 358.72, *p* < 0.001, *ΔR*^2^ = 0.054]. Two interaction terms were significant, attesting, respectively, that (1) the relationship between psychopathy and moral disengagement was significantly moderated by trust in known others, and (2) the relationship between Machiavellianism and moral disengagement was significantly moderated by trust in unknown others. We applied conventional procedures for computing simple slopes of psychopathy and Machiavellianism on moral disengagement at one standard deviation above and below the mean of trust in known or unknown other. Results showed that the relationship between psychopathy and moral disengagement was significant both when trust in known others was low or high, being (unexpectedly) higher in this latter case than in the former (see Figure 3A). Likewise, Machiavellianism (Figure 3B) was significantly associated with moral disengagement when trust in unknown others was low, and marginally when it was high.

#### Trust in Known Others

##### Hypothesized results

**FIGURE 4:**
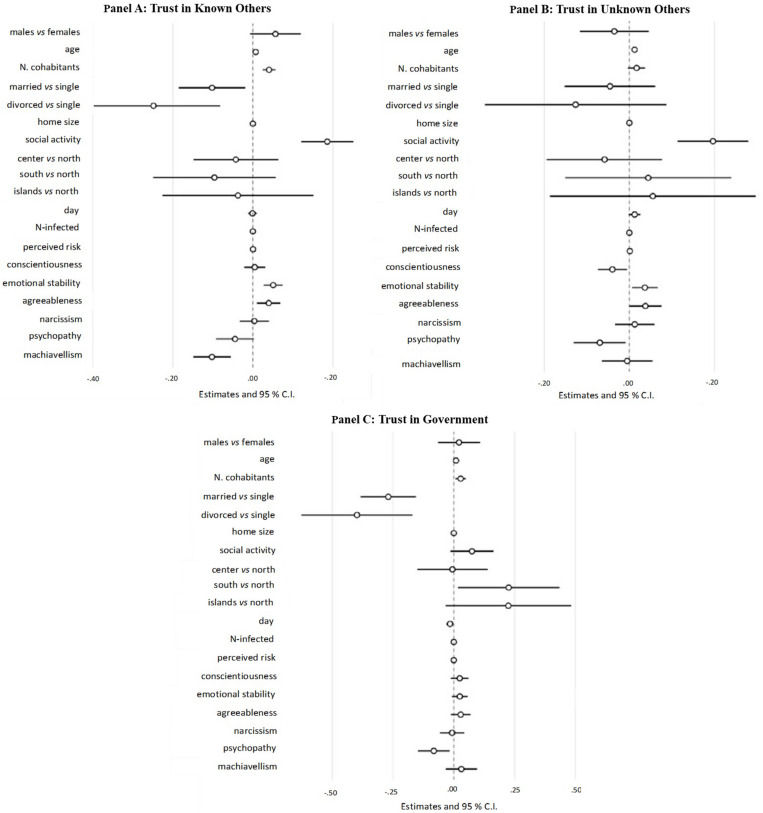
Results from the generalized social trust mediator model. The spheres represent regression coefficient estimates and the bars represent 95% confidence intervals (CIs). **(A)** Trust in known others. **(B)** Trust in unknown others. **(C)** Trust in Government. Trust k.o., trust in known others; Trust u.o., trust in unknown others; Trust go, trust in Government.

As shown in Figure 4A, emotional stability and agreeableness were significant positive predictors of trust in known others, whereas Machiavellianism was a negative predictor.

##### Not hypothesized results

Among covariates, social activity and number of cohabitants positively predicted trust in known others. Lower levels of trust in known others were found among married and divorced people than in single.

##### Model fit

The model explained a significant [*F*(19, 1500) = 11.11, *p* < 0.001] and moderate proportion of variance (*R*^2^ = 0.12).

#### Trust in Unknown Others

##### Hypothesized results

As shown in Figure 4B, emotional stability and agreeableness significantly and positively predicted trust in unknown others, whereas conscientiousness and psychopathy resulted in significant negative predictors.

##### Not hypothesized results

Among covariates, social activity and age significantly predicted trust in unknown others.

##### Model fit

The model explained a significant [*F*(19, 1500) = 7.928, *p* < 0.001] and moderately low proportion of variance (*R*^2^ = 0.09).

#### Trust in Government

##### Hypothesized results

As shown in Figure 4C, psychopathy was the only personality trait that predicted significantly trust in Government (with a negative association).

##### Not hypothesized results

Among covariates, higher levels of trust were found in the south compared to the north, in older people, and in people living with more cohabitant, and lower levels were found in married or divorced than in single people. Interestingly, trust in Government showed a slight decline with the passing of days.

##### Model fit

The model explained a significant [*F*(19, 1500) = 4.743, *p* < 0.001] and low proportion of variance (*R*^2^ = 0.04).

### Step 2. Outcome Models

#### Social Distancing

##### Hypothesized results

**FIGURE 5:**
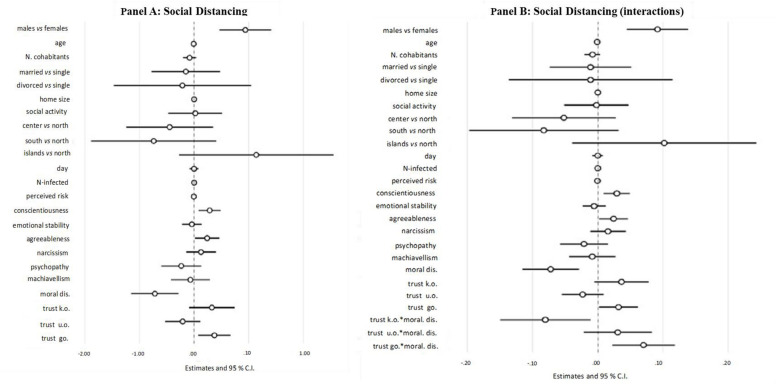
Results from the social distancing outcome model. The spheres represent regression coefficient estimates and the bars represent 95% confidence intervals (CIs). **(A)** Social distancing. **(B)** Social distancing (interactions). Trust k.o., trust in known others; Trust u.o., trust in unknown others; Trust go., trust in Government.

Figure 5A shows results for the prediction of social distancing. As hypothesized, moral disengagement and trust in Government were significant predictors of social distancing. Specifically, the relationship between moral disengagement and social distancing was negative and that of trust in Government was positive.

##### Not hypothesized results

However, also the personality traits of agreeableness and conscientiousness were significant and positive predictors of social distancing. Taken together, these results suggested partial mediation. Among covariates, more social distancing behaviors were performed by females.

##### Model fit

The model explained a significant [*F*(23, 1496) = 4.675, *p* < 0.001] and (low) proportion of variance (*R*^2^ = 0.07).

##### Hypothesized moderations

The interaction of moral disengagement with social trust significantly improved the fit of the model [*F*(3, 1493) = 269.56, *p* < 0.001, *ΔR*^2^ = 0.054]. As shown in Figure 5B, both trust in known others and trust in Government moderated the negative relationship between moral disengagement and social distancing. Results (Figure 3C) showed that the relationship between moral disengagement and social distancing was not significant at low levels, but significant at high levels of trust in known others. On the contrary, moral disengagement was significantly and negatively associated with social distancing when trust in Government was low but not for high trust in Government (Figure 3D).

#### Total Exits

##### Hypothesized results

**FIGURE 6:**
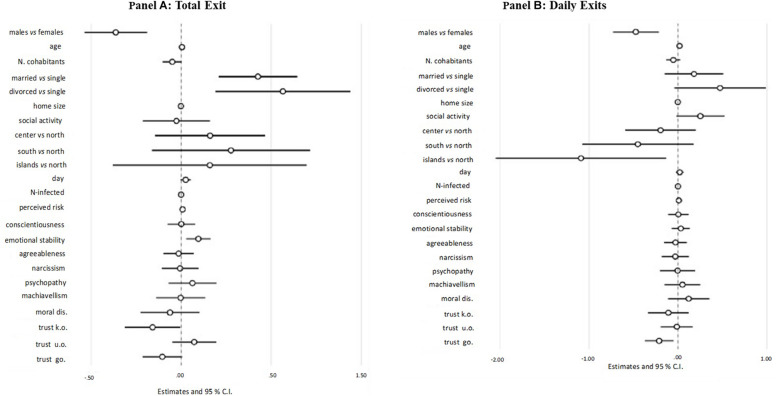
Results from the total exit and daily exits outcome model. The spheres represent regression coefficient estimates and the bars represent 95% confidence intervals (CIs). **(A)** Total exit. **(B)** Daily edits. Trust k.o., trust in known others; Trust u.o., trust in unknown others; Trust go., trust in Government; Moral. dis., moral disengagement.

Figure 6A shows results for the prediction of total exits. Among personality traits, emotional stability was significantly and positively associated with total number of exits. Accordingly, there was a 10% increase in the number of exits for any point increase in emotional stability. Trust in known others was, instead, significantly and negatively related to total number of exits (i.e., 15% less).

##### Not hypothesized results

Among covariates, we found that males, as well as married and divorced people, reported a significantly higher number of home exits since the beginning of the quarantine regime (10, 53, and 76% more exits, respectively) than females and singles. Finally, home size was significantly and negatively linked to the total number of exits (about 1% less), and people with a higher self-perceived risk to contract the infection reported a significantly higher number of exits from home (about 1% more).

##### Model fit

The model explained a moderately low proportion of variance (*R*^2^ = 0.09; residual deviance = 10,672.76, *df* = 1496). Adding the hypothesized interactions with moral disengagement did not significantly improve model fit [*Δr.dev* (3) = 52.05, *p* = 0.24, *ΔR*^2^ = 0.004].

#### Average Daily Exits

##### Hypothesized results

Figure 6B shows results for the prediction of daily exits. Among psychological variables, only trust in Government was significantly linked to average exits per day. People reporting more trust in Government stated a 19% less exit.

##### Not hypothesized results

Females reported a significant number of daily exits, about 66% lower than males, whereas people living in the two major islands reported a significant number of daily exits, about 66% lower than people living in the north. Finally, age (2%) and the subjective perception of the infection risk (1%) significantly predicted a significantly higher number of exits per day.

##### Model fit

The explained variance was low (*R*^2^ = 0.04; residual deviance = 673.76, *df* = 1496). Adding the interactions did not improve model fit [*Δr.dev* (3) = 14.821, *p* = 0.07, *ΔR*^2^ = 0.004].

### Implied Conditional Indirect Effects

Results regarding conditional indirect effects of personality traits were presented in Table 2 and Supplementary Table S7.

**TABLE 2:** Estimated conditional indirect effects.

		Moral disengagement Social distancing						Trust in government Social distancing					
		
		Em. stability		Narcissism		Psychopathy		Em. stability	Narcissism		Psychopathy		

Trust in known others		β	CI	β	CI	β	CI	β	CI	β	CI	β	CI
	Average	**0.002**	**0.0004, 0.004**	**−0.003**	**−0.006, −0.0004**	**−0.013**	**−0.022, −0.005**	**0.002**	**0.0004, 0.004**	**−0.003**	**−0.006, −0.0004**	**−0.013**	**−0.023, −0.005**
	High	**0.004**	**0.001, 007**	**−0.005**	**−0.0103, −0.0009**	**−0.029**	**−0.047, −0.013**	0.000	−0.001, 0.003	−0.001	−0.004, 0.002	−0.004	−0.019, 0.010
	Low	0.001	−0.001, 0.003	−0.001	−0.004, 0.002	−0.003	−0.011, 0.004	**0.004**	**0.0009, 0.007**	**−0.005**	**−0.011, −0.001**	**−0.015**	**−0.026, −0.007**

		**Trust in known others Total exits**						**Trust in government Daily exits**					
		**Emotional stability**		**Agreeableness**		**Machiavellianism**		**Psychopathy**					

		**β**	**CI**	**β**	**CI**	**β**	**CI**	**β**		**CI**			

	Average	**−0.008**	**−0.018, < 0.001**	−0.006	−0.016, < 0.001	**0.016**	**0.002, 0.032**	**0.017**		**0.001, 0.049**			

*Significant indirect effects are in boldface; Em. Stability, emotional stability.*

#### Moral Disengagement

##### Hypothesized indirect effects

The conditional indirect effects of the personality traits of emotional stability, narcissism, and psychopathy on social distancing through moral disengagement were significant only when (1) trust in known others was mean or high and (2) trust in Government was mean or low. Under these circumstances, people with high scores on emotional stability and low scores on narcissism or psychopathy practiced more social distancing. The first and second stage moderated indirect effect of Machiavellianism on social distancing through moral disengagement was, finally, significant only when trust in Government and in known others were at average levels (see Supplementary Table S8).

##### Not hypothesized indirect effects

Likewise, being female and the number of infected people indirectly predicted more social distancing through moral disengagement when trust in known others and Government were, respectively, high and low. An opposite pattern was found for being married or divorced and for the day of responding: the effect of those variables on social distancing through moral disengagement was negative when trust in known others and Government were, respectively, high and low.

#### Trust in Known Others

##### Hypothesized indirect effects

Among personality traits, trust in known others significantly mediated the relationship of emotional stability and Machiavellianism with total number of exits (but not of agreeableness). The indirect contribution of conscientiousness was negative (less exits), but positive for Machiavellianism (more exits).

##### Not hypothesized indirect effects

Age, number of cohabitants, and social activity indirectly predicted less exits from home, whereas being married or divorced indirectly predicted more exits from home.

#### Trust in Unknown Others

This variable did not significantly mediate any hypothesized or not hypothesized relationship.

#### Trust in Government

##### Hypothesized indirect effects

Trust in Government significantly mediated the relationship between psychopathy and average daily exits from home. This indirect effect was positive, thus suggesting a positive indirect contribution (more average daily exits).

##### Not hypothesized indirect effects

Living in the south and being married or divorced (compared to being single) all resulted in significant and positive indirect effects. On the contrary, age and number of cohabitants resulted in negative indirect effects. Accordingly, older and people living with more cohabitants reported more daily exits from home.

## Discussion

Coping with a pandemic outbreak is not something to which people can be psychologically prepared, or even conceived. Understandably, the Government efforts to contain the spread of COVID-19 in Italy have mostly dealt with a responsive but mostly unprepared population. Despite that, citizens’ efforts to comply with new regulation imposed on common life habits have been enormous and evidenced by momentous social initiative (such as #I stay at home, and similar others). In spite of generally intense and often heroic efforts, not everybody conformed to the imposed rules completely. Much worse, many people were caught violating quarantine or social distancing rules. Why?

As social scientists, we tried to understand these violations using well-established theoretical models based on the implicative construct of moral disengagement, coupled with expectations based on individual differences and characteristics of social environments embedding the individuals. The above theoretical framework has been fruitfully suited for predicting other kinds of rule-breaking behaviors (see, for example, Caprara et al., 2013, 2014). Results from this study suggest that this model can also be useful for a general understanding of people’s behaviors during the COVID-19 outbreak, although several qualifications are necessary.

For example, the mediating role of moral disengagement on the relationship between personality traits and rule-respecting behaviors was confirmed only for social distancing. Moral disengagement has nothing to say about compliance with the stay-at-home order that was instead predicted by GST and, in particular, by trust in Government and in (known) others. Importantly, the relationship of conscientiousness and agreeableness with rule-respecting behaviors was only partly mediated, and, for emotional stability, the relationship was inverse to that expected. Finally, GST emerged as a powerful gatekeeper governing moral disengagement, although not in the expected direction, and acted as a mediator itself. Below, we discuss our major findings in detail, explaining when they deviate from our original predictions and when not, and clarifying why we believe our results have much to offer to the scientific debate.

### Moral Disengagement

The role of moral disengagement as an individual’s specific adaptation working as a mediator of the link between basic traits and behaviors was supported only for the relationship between emotional stability, narcissism, psychopathy, and social distancing. Incidentally, an interesting finding is that all the members of the dark triad showed stronger associations than conscientiousness and agreeableness with moral disengagement. This evidence suggests that observed variability in the construct of moral disengagement may be made up of more deviant individual differences than of normal personality features than previously believed (but see DeLisi et al., 2014; Fossati et al., 2014 for a similar point).

The lack of association between moral disengagement with total and average number of exits from home was instead unexpected. Moral disengagement was introduced as a close predictor of individuals’ enacted behavior (Bandura, 2016), and thus it seemed likely that it should affect morally imbued behaviors such as staying at home. It is likely that people consider (implicitly or not) going in and out from home a basic and long earned freedom. Moreover, staying at home or exiting may often become necessary in reason of a well-established sequence of daily chores (i.e., buying food supplies, etc.) or the habit to do outdoor activity (i.e., running, training, etc.). In sum, we speculate that disrespecting the stay-at-home order may ultimately not be perceived every time as akin to a moral transgression. Rather, it could be that remaining at home, also in front of a perceived right need to do things outside, may require a form of moral participation of a different kind by that captured (in negative) by moral disengagement. Of course, these all remain provisional hindsight speculations.

### Generalized Social Trust

Two components of GST, namely, trust in Government and trust in known others, played a major role in our model. The first mediated the relationship between the basic traits and social distancing, a result that sustains our reasoning that rule-respecting behaviors are promoted if individuals trust the authority that is enforcing them (Rudolph and Evans, 2005). Trust in Government was also the unique predictor of the average number of daily exits from home. This result is fully in line with the prominent role played by the national Government in the managing of the COVID-19 crisis, in terms of taking the necessary steps to reduce it and of appropriately communicating with the population. Finally, as we predicted, trust in Government helped to counteract the tendency of individuals high in moral disengagement to enact less social distancing behaviors. The more people felt close to their Government, the more they remained morally engaged in following the rules they enforced (Marien and Hooghe, 2011). This is an obvious finding, but probably one of the more important to take in mind, in times of crisis.

The role of trust in known others was not less important than that of trust in Government, given that it resulted in a significant (negative) predictor of the total number of exits from home, and a significant moderator of the relationship between personality traits and moral disengagement. In line with our expectations, the more people perceived their acquaintances as trustworthy, the more they tried to respect the stay-at-home order. This result is fully in line with the theoretical assumption that individuals are more willing to respect social rules if they perceive that others are going to do the same (Scholly et al., 2005). According to our model, trust in known others is fostered by emotional stability and agreeableness but is hindered by Machiavellianism; thus, it became a significant mediator of the relationship of these traits with total number of exits.

Probably more important (but contrary to our expectations) was the moderation of trust in known others on the relationship among moral disengagement and social distancing. Higher levels of trust in known others seemed to exert a kind of disinhibitory effect on moral disengagement tendencies. Accordingly, people living in an environment perceived as more rule respecting and reciprocating reported a higher recourse to mechanism of moral disengagement and thus to engage in social distancing less. On the contrary, people being more suspicious and confiding less in others’ goodwill reported to have more social distancing. We believe that this paradoxical aspect of trust can have at least two explanations. From one side, social distancing from known others may be perceived by individuals as impolite, given it is contrary to the warmth style of interpersonal relations. From another side, people may reduce social distancing with known others because familiarity may induce a sense of overconfidence in thinking that they are less likely to be infected (Siegrist et al., 2005).

Unexpectedly, trust in known others and trust in unknown others exert the same disinhibitory effect on psychopathy and Machiavellianism, respectively, increasing its relationship with moral disengagement when high. Like they do with trust that others place in them, people high in psychopathy and Machiavellianism use their own feeling of trust in other people as a signal that others are more or less exploitable. Likely, people high in psychopathy and Machiavellianism use trust as a kind of “gullibility compass,” informing on the degree of exploitability in a social system. Interestingly, trust in known others and unknown others exerted opposite moderational effects on the indirect relationship between Machiavellianism with social distancing, canceling out each other.

### Basic Personality Traits

Contrary to our expectations, the relationship between basic personality traits and rule-respecting behaviors was, in most cases, only partially mediated. Indeed, conscientiousness and agreeableness resulted in a direct and positive relationship with social distancing, while emotional stability also directly predicted the total number of exits, but (unexpectedly) in a positive manner. It is likely that this effect may reflect a more resistance to the distress ingenerated by the need to cope with the possibility to encounter infected people outside, or a resulting type of overconfidence, but we have no further argument to corroborate this claim. Accordingly, this effect may reflect a sort of overconfidence. Interestingly, the effects of the dark traits all became completely mediated by moral disengagement. In light of these results, it seems likely that moral disengagement captures the best personality characteristics assessed by the dark triad, further reinforcing the idea that the dark triad and the normal big five dimensions capture different personality characteristics (Vize et al., 2020).

### Covariates

As presented in the main text, we found several significant effects of covariates. We are not going to discuss them in full detail here, given that we examined them mostly in an exploratory manner and many of them are in line with previous studies (i.e., the relationship between gender and moral disengagement; see Caprara et al., 2013, 2014). Two of them, however, seemed particularly interesting. The first attested a significant and negative relationship between the total number of infected people reported in a day and moral disengagement, and the second was the positive relationship between the days passed since the outbreak beginning and the levels of moral disengagement. These covariates resulted, respectively, in a positive (i.e., more) and a negative (i.e., less) indirect relationship with social distancing. We believe that considering the role played by these important elements of the social environment may be useful to promote the respect of rules.

### Limitations

This study has several limitations, including the use of short and exclusively self-report measures. However, reducing the time necessary to fill out the questionnaire appeared necessary not only in order to increase participation but also for reducing the burden on participants that were already distressed by the unusual situation. Whereas several proofs of the validity of these measures have been published, we believe that the low construct coverage and, in some cases, the low reliability of these instruments may have contributed to lower the size of the observed relationships. Indeed, the explained variance in the outcome variables was moderately low. These latter suggest that our conclusions should not be overstated. Moreover, the cross-sectional nature of the study prevents considerations about causality. Finally, and most importantly, not all hypotheses we stated were confirmed, and many results were opposite to our expectations. Whereas in hindsight they seemed fully reasonable and informative, we caution the reader to embrace our conclusion critically. We believe that the exceptionality of the situation makes our results specific to a certain social context and to a specific historical period. Our study should be considered akin to a social experiment, which we hope will never be replicated.

## Conclusion

Summarizing our study suggests that moral disengagement and social trust can be considered important elements to consider for promoting rule-respecting behaviors in times of emergency. Moral disengagement, for example, can be counteracted by taking some necessary steps suggested by social cognitive theory (Bandura, 2016). Among the components of GST, our results suggest that high level of trust in Government are ever beneficial, whereas average levels of trust in others (known or not) can be generally desirable, but somehow open the way to dysfunctional systemic effects. Having said that, if considered with the necessary qualifications, our results have the potential to contribute to the understanding of the determinants of rule-respecting behaviors during the early COVID-19 outbreak in Italy.

Indeed, whereas our results should not be overstated, they should not be understated as well. Whereas the size of associations was generally low, it is likely that decreasing people’s moral disengagement or increasing GST (or both) may lead to an accumulation of the effects of these constructs on rule-respecting behaviors to meaningful increases in these latter over time. Our point is that although the effects of decreasing moral disengagement or increasing GST may be relatively small for each single person, their cumulative implication for the society at large can be quite large. A similar point has been already made by Erol and Orth (2011) with regard to the (small) effects of life outcomes on self-esteem, and it is routinely redone regarding the (small) effects of lifesaver drugs, such as aspirin. Another point is that the effect of the quarantine regime may have induced a “strong situation effect,” leading to a reduction of the effect of individual differences, and thus of their association with rule-respecting behavior. Likely, in more liberal regime (i.e., the ongoing “reopening phases”), individuals’ behavior may be more variable on a single individual basis and thus more linked to ones’ own individual differences.

In sum, we recommend that the Government make a reasoned investment in civic education programs or, more generally, in all those interventions that may increase civic engagement (the contrary of civic disengagement) and GST at several levels. We can anticipate that the gain will not be great at the beginning but will pay in the end. By stating this, we implicitly suggest that, in the short run, external constrain and law-enforced rules may be more effective in reducing these behaviors, but a dual strategy centered on short-term objective and long-term goal may likely be more effective.

Of course, studies should go on in individuating factors that may sustain people engaging with rule-respecting behaviors. For this enterprise to become successful, we recommend that researchers use a preregistered analytic plan and make their data open to the scientific community, so that cumulative reliable knowledge can be built. The COVID-19 outbreak represents a unique opportunity for social science to effectively and timely contribute to improving the well-being of our contemporaneous society.

## Data Availability Statement

The datasets presented in this study can be found in online repositories. The names of the repository/repositories and accession number(s) can be found here: https://osf.io/dkbpj/?view_only=196be18f7b454e0a84799ebdb91129f3.

## Ethics Statement

The studies involving human participants were reviewed and approved by Sapienza Department of Psychology Internal Review Board. The patients/participants provided their written informed consent to participate in this study.

## Author Contributions

GA and LF conceived the study. GA ran the analyses and drafted the manuscript. GA, LF, EC, GC, and LA collected the data and commented on the manuscript. MT drafted and commented on the manuscript. All authors contributed to the article and approved the submitted version.

## Conflict of Interest

The authors declare that the research was conducted in the absence of any commercial or financial relationships that could be construed as a potential conflict of interest.

## Footnotes

1 https://aspredicted.org/blind.php?x=f37ni8

2 http://opendatadpc.maps.arcgis.com/apps/opsdashboard/index.html#/b0c68bce2cce478eaac82fe38d4138b1
