# Supplementary material for: Moral Disengagement and Generalized Social Trust as Mediators and Moderators of Rule-Respecting Behaviors During the COVID-19 Outbreak
Source: Front Psychol. 2020 Aug 27;11:2102. doi: 10.3389/fpsyg.2020.02102 (PMC7481453; doi:10.3389/fpsyg.2020.02102)
Supplement: Supplementary file 1 [file Data_Sheet_1.docx]

Supplementary Materials

Moral Disengagement and Generalized Social Trust as Mediators and Moderators of Rule Respecting Behaviors During COVID-19 Outbreak

**Measures used in the study**

Below we present in full detail the instruments used in this study and the tables referenced in the main text of the manuscript. We provide a description of each scale along with its psychometric properties. The items content of each scale is presented in the respective tables (see Table S1-S5). The reliability of each scale was estimated using McDonald’s ω (McDonald, 1999), following suggestions by Trizano-Hermosilla and Alvarado (2016).

**Big Five**. Personality traits were assessed with the Italian version (Chiorri, Bracco, Piccinno, Modafferi, & Battini, 2015) of the Ten Item Personality Inventory (TIPI; Gosling, Rentfrow, & Swann, 2003). The TIPI consists of adjectives and descriptors taken from other well-established Big Five instruments. The common stem is ‘I see myself as:’, and each item include two adjectives or descriptors (e.g. ‘Extraverted, enthusiastic’ or ‘Anxious, easily upset’) to which participants have to indicate their level of agreement, using a 7-point scale ranging from 1 (= strongly disagree) to 7 (= strongly agree). The ω coefficients were ω = .25 for agreeableness, ω = .45 for conscientiousness and ω = .57 for emotional stability.

**Dark Triad**. We measured dark triad traits (see Table S2) with the Italian version (Schimmenti et al., 2019) of the Dark Triad Dirty Dozen (DTDD; Jonason & Webster, 2010). The DTDD is composed by 12 items, four per each dark triad trait (e.g. ‘I tend to manipulate others to get my way’ for Machiavellianism; ‘I tend to want others to admire me’ for narcissism, ‘I tend to be callous or insensitive’ for psychopathy). The response format was a 5-point scale ranging from 1 (= strongly disagree) to 5 (= strongly agree), and the ω coefficients were ω = .87 for Machiavellianism, ω = .70 for psychopathy and ω = .82 for narcissism.

**Moral disengagement**. We assessed moral disengagement (see Table S3) using a reduced version of the Civic Moral Disengagement scale (CMD; Caprara et al., 2009). Our reduced version consisted of 8 items, one for each moral disengagement mechanism, selected from the original 32-item version. The criterion was to select a marker item (i.e., the item with highest factor loading) for each of the eight dimensions of the moral disengagement.

This instrument was developed on a previous dataset composed by 227 participants (33% males, *M* = 28.90 e (*SD* = 1.69), who agreed in taking part to another project focused on moral civic disengagement some years ago. These participants answered to both the original scale and the shortened instrument. This allowed us to correlate the individuals’ average score on the eight items used in this study with the average score of all 32 original items with and without the including the 8 items included on the shortened instrument. The correlations of the shortened scale with the longer one were *r* = .92 (with the full measure, composed by 32 items), and *r* = .88 (with a version composed only with the 24 items not included in the shortened version ) respectively. In addition, we performed a CFA (the estimator was WLSMV) on both the previous and the present dataset, in order to evaluate the internal structure of this reduced version of the scale. Each model provided good fit to the data: (1) WLSMV-Based *χ^2^*(20, *N* = 227) = 60.809, *p* < .001; CFI = .968; RMSEA = .096, 90% CI [.069, .124], *p* < .05 for the model tested on the previous dataset; (2) WLSMV-Based *χ^2^*(20, *N* = 1520) = 180.212, *p* < .001; CFI = .964; RMSEA = .073, 90% CI [.063, .083], *p* < .001 for the model tested on the OCG-COVID19 data. The reliability of this scale was good in both the previous and the present dataset (ω = .83 and ω = .74, respectively), and, overall, all the analyses performed resulted acceptable (for further details see Table S3).

**Generalized social trust**. According to the OECD (Gonzales & Smith, 2017), ‘trust in known others’, ‘trust in unknown others’ and trust in Government are three of the most important components of generalized social trust. We asked the participant to indicate how much trust they had in a number of groups and institutions, and they rated each item using a 5-point scale ranging from 1 (= no trust at all) to 5 (= complete trust). As presented in Table S4, items for trust in known others referred to family, friends, neighborhood, colleagues and superiors. For trust in unknown others, we used items referring to unknown people from the same country of the participants and unknown people from different countries. Finally, trust in Government was measured by means of 4 items referring to the Italian Government, the Italian Prime Minister Giuseppe Conte, the President of the Italian Republic Sergio Mattarella and the European Union. The ω coefficients were ω = .71 for trust in known others, ω = .91 for trust in unknown others and ω = .83 for trust in Government.

**Social distancing**. We evaluated the degree to which the participants respected the social distancing rule using a 5-item scale created *ad hoc* for this study. Table S5 presents the text of each item and the psychometric properties of the scale. Participants rated each item using a 5-point scale ranging from 1 (= strongly disagree) to 5 (= strongly agree), and the reliability (ω = .58) was acceptable.

**Social activities**. We asked participants to indicate the frequency with which they used to engage in social activities before the COVID-19 outbreak. This scale was composed *ad hoc* for the study. Participants rated each of the 8 items (see Table S6), using the following 5-point scale: never (= 1), sometimes a year (= 2), sometimes a month (= 3), sometimes a week (= 4) and several times a week (= 5). This scale provided a good reliability (ω = .72).

Table S1. Psychometric Properties of the Ten Item Personality Inventory

| Item | Text | *M* | *SD* |
| --- | --- | --- | --- |
| *Extraversion* | | 4.01 | 1.43 |
| TIPI_1 | Extraverted, enthusiastic. | 4.50 | 1.72 |
| TIPI_6 | Reserved, quiet. (R) | 4.48 | 1.65 |
|  |  |  |  |
| *Agreeableness* | | 5.12 | 1.10 |
| TIPI_2 | Critical, quarrelsome. (R) | 3.47 | 1.78 |
| TIPI_7 | Sympathetic, warm. | 5.70 | 1.08 |
|  | |  |  |
| *Conscientiousness* | | 5.29 | 1.16 |
| TIPI_3 | Dependable, self-disciplined. | 5.81 | 1.11 |
| TIPI_8 | Disorganized, careless. (R) | 3.22 | 1.74 |
|  | |  |  |
| *Emotional Stability* | | 4.45 | 1.40 |
| TIPI_4 | Anxious, easily upset. (R) | 3.86 | 1.83 |
| TIPI_9 | Calm, emotionally stable. | 4.75 | 1.50 |
|  |  |  |  |
| *Openness* | | 4.50 | 1.08 |
| TIPI_5 | Open to new experiences, complex. | 5.44 | 1.23 |
| TIPI_10 | Conventional, uncreative. (R) | 4.45 | 1.55 |

*Note*. *M* = mean; *SD* = standard deviation; (R) = item reverse.

Table S2. Psychometric Properties of the Dark Triad Dirty Dozen.

| Item | Text | *M* | *SD* | *r_tt_* |
| --- | --- | --- | --- | --- |
| *Machiavellianism* | | 1.69 | .78 |  |
| DTDD_1 | I tend to manipulate others to get my way. | 1.68 | .91 | .84 |
| DTDD_2 | I have used deceit or lied to get my way. | 1.74 | .95 | .79 |
| DTDD_3 | I have use flattery to get my way. | 1.81 | .98 | .76 |
| DTDD_4 | I tend to exploit others towards my own end. | 1.55 | .82 | .71 |
|  | |  |  |  |
| *Psychopathy* | | 1.88 | .74 |  |
| DTDD_5 | I tend to lack remorse | 2.04 | 1.09 | .34 |
| DTDD_6 | I tend to be unconcerned with the morality of my actions. | 1.56 | .84 | .46 |
| DTDD_7 | I tend to be callous or insensitive. | 1.83 | .99 | .58 |
| DTDD_8 | I tend to be cynical. | 2.07 | 1.16 | .51 |
|  | |  |  |  |
| *Narcissism* | | 2.51 | .93 |  |
| DTDD_9 | I tend to want others to admire me. | 2.79 | 1.22 | .71 |
| DTDD_10 | I tend to want others to pay attention to me. | 2.95 | 1.16 | .69 |
| DTDD_11 | I tend to seek prestige or status. | 2.34 | 1.19 | .63 |
| DTDD_12 | I tend to expect special favors from others. | 1.95 | 1.05 | .54 |

*Note*. *M* = mean; *SD* = standard deviation; *r_tt_* = item-total scale-score-corrected correlation coefficient.

Table S3. Psychometric Properties of the Civic Moral Disengagement-Reduced Version.

| Item | Text |  | Previous Study *N* = 227 | | | |  | Present Study *N* = 1520 | | | |
| --- | --- | --- | --- | --- | --- | --- | --- | --- | --- | --- | --- |
|  |  |  | *M* | *SD* | *r_tt_* | *λ* |  | *M* | *SD* | *r_tt_* | *λ* |
| CMD_1 | For the advance of science, it is lawful to use humans as ‘‘guinea pigs” even in high risk experiments |  | 1.38 | .76 | .43 | .53 |  | 1.85 | 1.02 | .29 | .33 |
| CMD_2 | Fraud in economic transactions is simply a ‘‘strategic distortion” |  | 1.43 | .76 | .74 | .88 |  | 2.26 | 1.13 | .41 | .43 |
| CMD_3 | Citizens who litter the streets should not be severely persecuted since industry produces much more serious pollution |  | 1.35 | .71 | .64 | .78 |  | 1.60 | .83 | .46 | .57 |
| CMD_4 | If someone loses control during a brawl, he/she is not completely responsible for the consequences of his/her actions |  | 1.55 | .78 | .57 | .66 |  | 2.03 | .99 | .52 | .59 |
| CMD_5 | There is no sense in blaming individuals who evade a rule when everybody else does the same thing |  | 1.49 | .78 | .61 | .72 |  | 1.73 | 1.00 | .67 | .80 |
| CMD_6 | Evading taxes cannot be considered reprehensible considering the squandering of public money |  | 1.67 | .99 | .54 | .65 |  | 1.62 | .92 | .61 | .74 |
| CMD_7 | Victims generally have trouble staying out of harm’s way |  | 1.55 | .79 | .69 | .82 |  | 1.77 | .93 | .55 | .64 |
| CMD_8 | Rivals deserve being humiliated and maltreated |  | 1.31 | .67 | .64 | .80 |  | 1.46 | .74 | .54 | .65 |

*Note*. *M* = mean; *SD* = standard deviation; *r_tt_* = item-total scale-score-corrected correlation coefficient; *λ* = standardized factor loading.

Table S4. Psychometric Properties of the Generalized Social Trust Scale.

| Item | Text | | *M* | *SD* | *r_tt_* |
| --- | --- | --- | --- | --- | --- |
| *Trust in Known Others* | | |  |  |  |
| TRUSTKO_1 | | Your family members | 4.38 | .81 | .44 |
| TRUSTKO_2 | | Your friends | 3.83 | .80 | .59 |
| TRUSTKO_3 | | Your neighbors | 2.54 | .96 | .55 |
| TRUSTKO_4 | | Your work or study colleagues | 3.10 | .89 | .68 |
| TRUSTKO_5 | | Your superiors (if you have them, otherwise your professors) | 3.02 | .99 | .53 |
|  | | |  |  |  |
| *Trust in Unknown Others* | | |  |  |  |
| TRUSTUO_1 | | People you don't know who are of your nationality | 2.32 | .80 | .87^#^ |
| TRUSTUO_2 | | People you don't know who are of another nationality | 2.29 | .79 | - |
|  | | |  |  |  |
| *Trust in Government* | | |  |  |  |
| TRUSTGO_1 | | The Italian Government | 2.82 | .91 | .79 |
| TRUSTGO_2 | | The Prime Minister Giuseppe Conte | 3.14 | .96 | .81 |
| TRUSTGO_3 | | The President of the Italian Republic Sergio Mattarella | 3.35 | 1.11 | .77 |
| TRUSTGO_4 | | The European Union | 2.43 | .96 | .51 |

*Note*. *M* = mean; *SD* = standard deviation; *r_tt_* = item-total scale-score-corrected correlation coefficient; # = for Trust in Unknown Other scale was not possible to compute the item-total scale-score-corrected correlation coefficient, therefore here is reported the correlation between the two items.

Table S5. Psychometric Properties of the Social Distancing Scale.

| Item | Text | *M* | *SD* | *r_tt_* |
| --- | --- | --- | --- | --- |
| SocDis_1 | I keep the recommended distance from people and avoid crowded places. | 4.77 | .61 | .41 |
| SocDis_2 | I've limited my social interactions. | 4.47 | .98 | .33 |
| SocDis_3 | I'm strictly following the guidelines issued by the Government. | 4.63 | .66 | .35 |

*Note*. *M* = mean; *SD* = standard deviation; *r_tt_* = item-total scale-score-corrected correlation coefficient; *λ* = standardized factor loading.

Table S6. Psychometric Properties of the Social Activity Scale.

| Item | Text | *M* | *SD* | *r_tt_* |
| --- | --- | --- | --- | --- |
| SocActivity_1 | Theatre | 1.67 | .75 | .33 |
| SocActivity_2 | Cinema | 2.47 | .83 | .54 |
| SocActivity_3 | Museums/Exhibitions | 2.07 | .68 | .46 |
| SocActivity_4 | Concerts | 1.84 | .65 | .57 |
| SocActivity_5 | Sporting shows | 1.79 | 1.00 | .25 |
| SocActivity_6 | Pub | 2.78 | 1.25 | .59 |
| SocActivity_7 | Restaurant | 3.09 | .86 | .58 |
| SocActivity_8 | Disco | 1.73 | .95 | .49 |

*Note*. *M* = mean; *SD* = standard deviation; *r_tt_* = item-total scale-score-corrected correlation coefficient.

**Additional Results**

Conditional indirect effects referred in the main text regarding covariates and results regarding Machiavellianism are presented in Table S7 and S8, respectively.

Table S7. Estimated Conditional Indirect Effects for Covariates

|  |  | Moral disengagement 🡪 Social distancing | | | | | | | | | |
| --- | --- | --- | --- | --- | --- | --- | --- | --- | --- | --- | --- |
|  |  | Sex | | Married | | Divorced | | Day | | N-Infected | |
| Trust in known others | | 𝛽 | C.I. | 𝛽 | C.I. | 𝛽 | C.I. | 𝛽 | C.I. | 𝛽 | C.I. |
|  | average | .005 | .0004, .011 | -.006 | -.014, -.0008 | -.017 | -.035,-.004 | -.001 | -.002, 0003 | .00 | <.001, <.001 |
|  | high | .008 | .0082, .017 | -.011 | -.023, -.002 | -.029 | -.056, -.009 | -.002 | -.004, -.0001 | .00 | <.001, <.001 |
|  | low | .002 | -.0023, .007 | -.002 | -.009, .003 | -.006 | -.022, .009 | .000 | -.002, .0006 | .00 | < .001,< -.001 |
|  |  | Trust in Government 🡪 Total exits | | | | | | | | | |
|  |  | Sex | | Married | | Divorced | | Day | | N-Infected | |
| Trust in known others | | 𝛽 | C.I. | 𝛽 | C.I. | 𝛽 | C.I. | 𝛽 | C.I. | 𝛽 | C.I. |
|  | average | .005 | .004, .010 | -.006 | -.014, -.001 | -.017 | -.035, -.005 | -.001 | -.002, -.0003 | .00 | < .001, < .001 |
|  | high | .001 | -.003, .006 | -.001 | -.008, .004 | -.004 | -.021, .011 | .000 | -.001, .0008 | .00 | < -.001, < .001 |
|  | low | .008 | .001, .017 | -.011 | -.023, -.002 | -.031 | -.057, -.011 | -.002 | -.004, -.0001 | .00 | < .001, < .001 |
|  |  | Trust in known others 🡪 Total exits | | | | | | | | | |
|  |  | Married | | Divorced | | Social activity | | N-cohabitants | | Age | |
| Trust in known others | | 𝛽 | C.I. | 𝛽 | C.I. | 𝛽 | C.I. | 𝛽 | C.I. | 𝛽 | C.I. |
|  | average | .02 | .001, .040 | .039 | .003, .090 | -.030 | -.030, -.004 | -.0064 | -.013, -.001 | -.001 | -.002, -.001 |
|  |  | Married | | Divorced | | N-cohabitants | |  |  |  |  |
| Trust in known others | | 𝛽 | C.I. | 𝛽 | C.I. | 𝛽 | C.I. |  |  |  |  |
|  | average | .057 | .012, .114 | .084 | .015, .178 | -.006 | -.014, < .001 |  |  |  |  |

Table S8. Estimated Conditional Indirect Effects for Machiavellianism

|  |  | Moral disengagement 🡪 Social distancing | | | | | |
| --- | --- | --- | --- | --- | --- | --- | --- |
|  |  | Machiavellianism | |  |  | Machiavellianism | |
| Trust k.o. | Trust u.o. | 𝛽 | C.I. | Trust go. | Trust u.o. | 𝛽 | C.I. |
| average | average | -.01 | -.013, -.002 | average | average | -.01 | -.013, -.002 |
| high | high | -.03 | -.067, .002 | high | high | .00 | -.023, .012 |
| low | low | .00 | -.012, .006 | low | low | -.02 | -.050, .011 |
| low | high | -.01 | -.026, .009 | low | high | -.03 | -.070, .003 |
| high | low | -.02 | -.048, .010 | high | low | .00 | -.016, .008 |

*Note.* Trust k.o. = Trust in known other; Trust u.o. = Trust in unknown others; Trust go. = Trust Government

**References**

Caprara, G. V., Fida, R., Vecchione, M., Tramontano, C., & Barbaranelli, C. (2009). Assessing civic moral disengagement: Dimensionality and construct validity. *Personality and Individual Differences*, *47*(5), 504-509. doi: 10.1016/j.paid.2009.04.027

Chiorri, C., Bracco, F., Piccinno, T., Modafferi, C., & Battini, V. (2015). Psychometric properties of a revised version of the Ten Item Personality Inventory. *European Journal of Psychological Assessment*, *31*(2), 109-119. doi: 10.1027/1015-5759/a000215

Gonzales, S., & Smith, C. (2017). The Accuracy of Measures of Institutional Trust in Household Surveys: Evidence from the OECD Trust Database. *OECD Statistics Working Paper*, *2017/11*, 1-38. doi: 10.1787/18152031

Gosling, S. D., Rentfrow, P. J., & Swann Jr, W. B. (2003). A very brief measure of the Big-Five personality domains. *Journal of Research in Personality*, *37*(6), 504-528. doi: 10.1016/S0092-6566(03)00046-1

Jonason, P. K., & Webster, G. D. (2010). The dirty dozen: A concise measure of the dark triad. *Psychological Assessment*, *22*(2), 420–432. doi:10.1037/a0019265

McDonald, R. P. (1999). *Test theory: A unified treatment*. Hillsdale, MI: Erlbaum.

Schimmenti, A., Jonason, P. K., Passanisi, A., La Marca, L., Di Dio, N., & Gervasi, A. M. (2019). Exploring the dark side of personality: Emotional awareness, empathy, and the Dark Triad traits in an Italian Sample. *Current Psychology*, *38*(1), 100-109. doi: 10.1007/s12144-017-9588-6

Trizano-Hermosilla, I., & Alvarado, J. M. (2016). Best alternatives to Cronbach's alpha reliability in realistic conditions: Congeneric and asymmetrical measurements. *Frontiers in Psychology*, *7*, 769-716. doi: 10.3389/fpsyg.2016.00769
